# Generalized Non-syndromic Hypertaurodontism With Delayed Eruption: A Case Report

**DOI:** 10.7759/cureus.62568

**Published:** 2024-06-17

**Authors:** Mrunali Deshkar, Sakshi P Kabra, Nilima R Thosar, Ramakrishna Yeluri

**Affiliations:** 1 Pediatric and Preventive Dentistry, Sharad Pawar Dental College and Hospital, Datta Meghe Institute of Higher Education and Research (Deemed to be University), Wardha, IND

**Keywords:** mixed dentition, indirect pulp capping, chronological age, delayed tooth eruption, taurodontism

## Abstract

Taurodontism is a rare dental anomaly defined by a change in tooth shape due to Hertwig's epithelial sheath not folding inward at the right horizontal level. It has a larger pulp chamber and a pulpal floor that is shifted apically, and the cementoenamel junction (CEJ) is not constricted. This condition is more frequently observed in permanent teeth than in primary teeth and can occur in a bilateral or unilateral manner, affecting any quadrant or group of teeth. This brief case report discusses a 14-year-old female patient who presented with complaints of decayed teeth in the lower right and left posterior regions of the jaw. Radiographic examination revealed the presence of non-syndromic taurodontism in both the deciduous teeth and their permanent successors. Dental management included oral prophylaxis, application of pit and fissure sealants, indirect pulp capping, and restoration with glass ionomer cement for the affected teeth.

## Introduction

Taurodontism is a morpho-anatomical alteration of the tooth's morphology that typically affects teeth with multiple roots [1]. While it is less common among modern people, taurodontism was a widespread anomaly discovered in Neanderthal humans [2]. A study conducted in Israel found that the incidence was 5.6%. According to reports, it is less than 1% in modern humans and 3% in apes, primitive people, and American Indians. Current case reports in dental literature suggest that taurodontism is becoming less uncommon and is no longer regarded as a rare anomaly among modern humans [3].

Taurodontism has been observed in primary and permanent teeth, with a prevalence ranging from <1% to 35%, depending on the population under investigation [4]. In comparison to the first molars, the second and third molars in the permanent dentition are affected more. There have been reports of autosomal dominant or recessive and X-linked inheritance patterns linked to taurodontism. There is a connection between taurodontism and the distal-less homeobox (*DLX3*) gene, which is expressed during root formation [5].

The etiology of taurodontism is unknown. The failure of Hertwig's epithelial sheath diaphragm to invade at the appropriate horizontal level is believed to be the cause. The distinctive characteristics include a wider pulp chamber, apical pulpal floor displacement, and no constriction at the cementoenamel junction (CEJ) level [1]. The majority of affected teeth are permanent molars and are commonly seen in males.

Taurodontism is most commonly seen as an independent anomaly, while it has been linked to some disorders, including tricho-dento-osseous syndrome, Klinefelter syndrome, and Down syndrome, and rare ones such as Smith-Magenis syndrome, Williams syndrome, McCune-Albright syndrome, and Van der Woude syndrome [6-8]. A preoperative radiograph is a useful diagnostic technique for taurodontism but uncommon and can help prevent unexpected issues over the course of successful endodontic therapy. Radiographic features that differentiate these teeth include extended pulp chambers and the downward displacement of root bifurcation or trifurcation zones. Unlike the occluso-cervical distance, the distance between the root bifurcation and the cementoenamel junction (CEJ) is notably greater. In terms of clinical appearance, the pulp chamber has a higher apico-occlusal height, which may give the impression that the access cavity is mistakenly perforated. Its rectangular shape is due to the absence of constriction at the CEJ [7].

Shaw categorized taurodontism into three types based on the extent of the apical displacement of the pulp chamber floor [9]. The pulp chamber is moderately enlarged in hypotaurodontism, at the expense of the roots. The pulp chamber is rather extensive in mesotaurodontism, but the roots stay short and distinct. The hallmark of hypertaurodontism is a pulp chamber that is either prismatic or cylindrical and reaches almost to the apex before splitting into two or four channels [9,10].

This case describes a 14-year-old female patient who presented with a delayed eruption of permanent teeth with generalized taurodontism in the maxillary and mandibular arches. The patient had no accompanying syndromes or medical conditions.

## Case presentation

**Figure 1:**
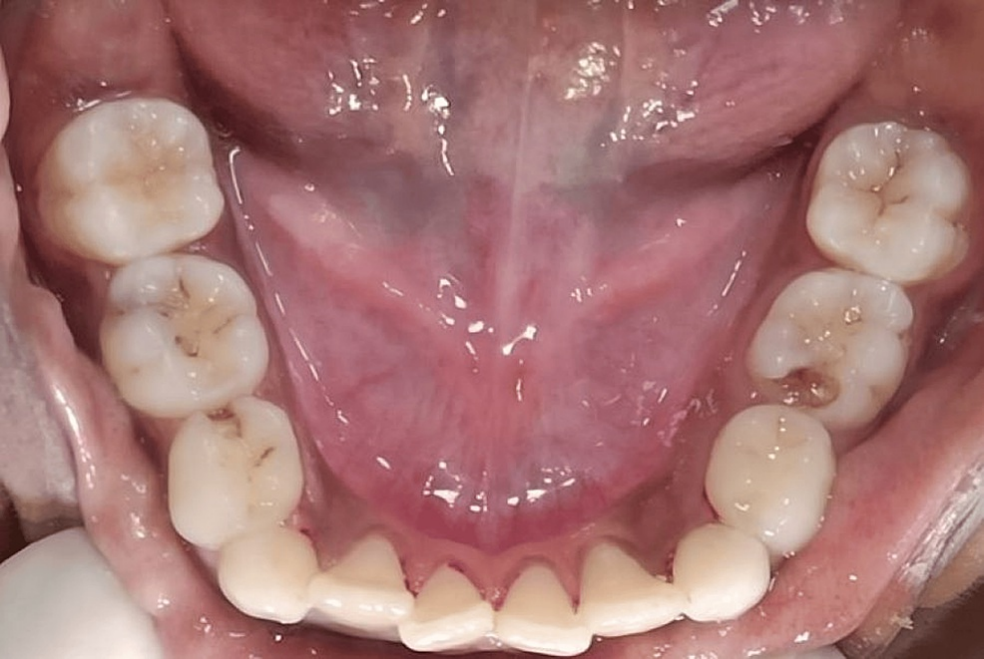
Preoperative image of 75 with deep proximal caries, 84 with proximal caries, and 85 with pit caries

A 14-year-old female reported at the Department of Pediatric and Preventive Dentistry, complaining of decay in the lower right and left molar region of the jaw as her main concern. The medical history of the patient was not significant. There was no family history of dental anomalies or prenatal or postnatal problems, according to the patient's mother. Based on a clinical assessment, the primary molar crowns had a normal shape and were associated with dental caries with respect to 84 and 75 (Figure 1).

**Figure 2:**
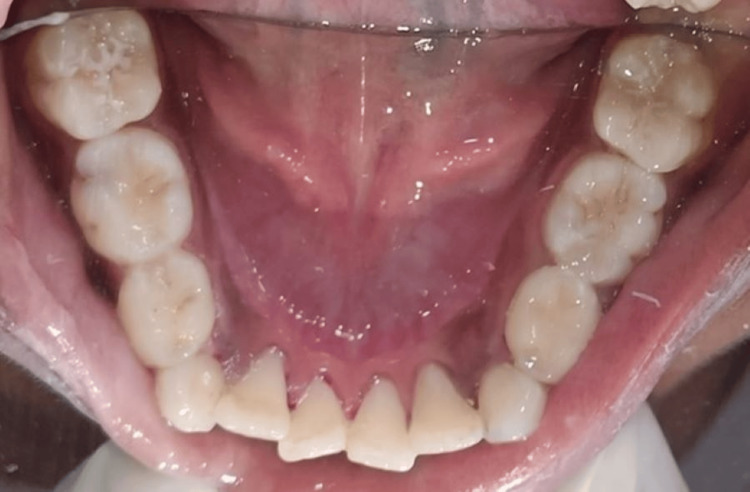
Postoperative image depicting indirect pulp capping with respect to 75, followed by GIC with 75 and 84, and pit and fissure sealant with permanent molars and preventive resin restoration with 85 GIC: glass ionomer cement

Because of a deep carious lesion present in tooth 75, it was advised to perform an intraoral periapical (IOPA) radiograph. The radiographic evaluation showed an enlarged pulp chamber with short roots and caries approaching the pulp with respect to 75 and dentinal caries with respect to 85. Radiographically, the crowns of molars were observed to be larger than their roots. Moreover, both the pulp chambers and root bifurcations were notably positioned significantly deeper below the cementoenamel junction (CEJ). The narrowing observed at the level of the CEJ was slight and not significant. Thus, the treatment of indirect pulp capping was carried out with respect to 75, and restoration with glass ionomer cement was carried out with respect to 84. In the preventive procedure, oral prophylaxis followed by pit and fissure sealant with permanent molars, preventive resin restoration with 85 and fluoride application with the upper and lower arch were done (Figure 2).

**Figure 3:**
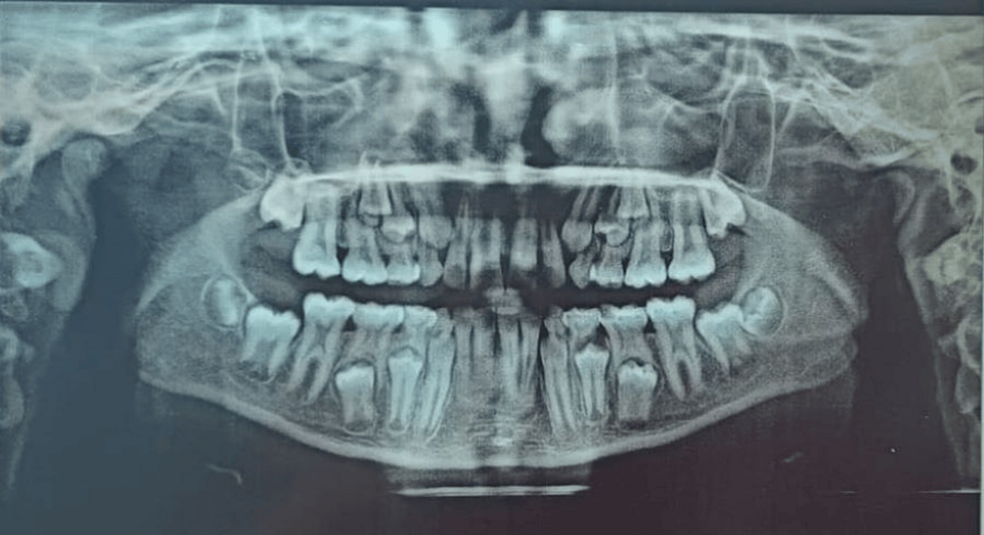
Orthopantomogram depicting generalized enlarged pulp chamber with short roots in all permanent and primary molars, and permanent anteriors showing open apex indicating delayed eruption Radiographic findings suggest the presence of a large pulp chamber and short roots with respect to the erupting permanent second molar, indicating hypertaurodontism.

In the current case report, the principle of Shifman and Chanannel [11] was used to diagnose taurodontism. Based on the radiographic evaluation, in order to exclude the possibility of taurodontism in other teeth, radiographic interpretation was performed on an orthopantomogram (OPG) and confirmed taurodontism in multiple teeth of the same child. Radiographic findings suggested the presence of a large pulp chamber and short roots with respect to the primary first and second molars of the right and left side of maxillary and mandibular arch teeth (54, 55, 16, 64, 65, 26, 75, 36, 84, 85, and 46) (Figure 3).

The upper central incisors had an open apex, indicating that two-thirds of the roots had formed. Analyzing the difference between the patient's chronological and dental age and clinico-radiographic evaluation, a diagnosis of generalized non-syndromic hypertaurodontism with delayed eruption was made. No other anomalies were noticed on OPG.

## Discussion

Witkop [12] defines a taurodont tooth as having larger pulp chambers and an apically displaced root trifurcation or bifurcation, which causes the pulp cavity's apico-occlusal width to lengthen and the tooth's cervical region to remain free of constriction. Taurodontism is observed in association with a number of well-known syndromes, including Down syndrome and Klinefelter syndrome, as well as a few medical conditions including hypophosphatasia. Newer theories about the pathophysiology of taurodontism have been reported by researchers. It is characterized by a distinctive formation sequence involving variations in the organization of epithelial cells within Hertwig's epithelial root sheath (HERS), delayed calcification of the pulpal chamber, and a varying presence of odontoblasts [1].

Taurodontism is often seen more in permanent teeth than in primary teeth, possibly due to differences in how it's diagnosed and variations across ethnicities. Permanent dentition commonly has a higher count of posterior teeth than primary dentition, and primary teeth may display unique root and crown shapes. These factors may account for the variations in tooth predominance between the two sets of teeth. According to reports, taurodontism affects roughly 0.3% of children and is detected in humans at a frequency ranging from 2.5% to 11.3% [13]. Moreover, maxillary molars (57%) exhibit a higher frequency of involvement compared to mandibular molars (43%), with taurodontic teeth being more frequently observed in the maxillary arches than in the mandibular arches. Hypotaurodontism, which has a 94% prevalence, is the most common kind of taurodontism seen [14].

The risk of pulp exposure possibly increased as to the clinical implications of taurodontism. With an increased risk of hemorrhage during access opening that could be mistaken for a perforation, endodontic treatment for teeth with taurodontism has thus been characterized as a complex and challenging technique [15]. In the present case, the patient's language and psychomotor development were age-appropriate. There was no syndrome that could have been connected to the case. Taurodontism was identified in the primary dentition through radiographic observations, and this characteristic was also noticeable in the succeeding permanent teeth, with delayed eruption.

## Conclusions

Due to its low prevalence, taurodontism needs to be properly diagnosed and treated with the aid of a comprehensive clinical and radiographic examination. Every dental professional should recognize this anomaly in dentistry and have a complete understanding of the identification and treatment of taurodontic teeth to avoid subsequent pain or endodontic failures. Appropriate identification is vital in assessing the treatment strategy and prognosis for taurodontic teeth.
